# 2,2,10-Trimethyl-2,3-dihydro­pyrano[2,3-*a*]carbazol-4(11*H*)-one

**DOI:** 10.1107/S1600536808033862

**Published:** 2008-10-22

**Authors:** Makuteswaran Sridharan, Karnam J. Rajendra Prasad, Aimable Ngendahimana, Matthias Zeller

**Affiliations:** a Department of Chemistry, Bharathiar University, Coimbatore 641 046, Tamil Nadu, India; b Department of Chemistry, Youngstown State University, One University Plaza, Youngstown, OH 44555, USA

## Abstract

The title compound, C_18_H_17_NO_2_, was prepared from 1-hydr­oxy-8-methyl­carbazole and 3,3-dimethyl­acrylic acid with trifluoro­acetic acid as the cyclization catalyst. Due to the –CMe_2_– group, the mol­ecule is not quite planar. The packing is dominated by the strong N—H⋯O hydrogen bonds and some weaker C—H⋯O and C—H⋯π inter­actions. π–π Stacking inter­actions [centroid–centroid separation = 3.806 (2) Å] join neighboring mol­ecules into loosely connected inversion dimers.

## Related literature

Knölker & Reddy (2002 ▶) report on the isolation of pyran­o­carbazoles from various plant species. Sridharan *et al.* (2007 ▶) describe the synthesis of compounds related to the title compound. Sridharan, Rajendra Prasad & Zeller (2008 ▶) report the structure of the 9-methyl derivative of the title compound. Sridharan, Rajendra Prasad, Ngendahimana *et al.* (2008 ▶) report the structure of the 10-*H* derivative of the title compound.
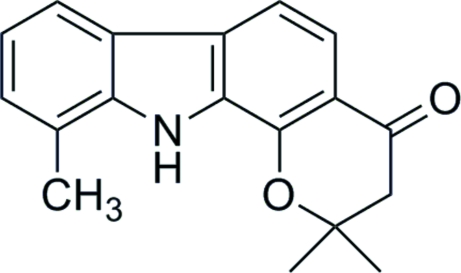


## Experimental

### 

#### Crystal data

C_18_H_17_NO_2_
*M* *_r_* = 279.33
Monoclinic, 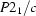

*a* = 12.9740 (16) Å
*b* = 9.4195 (12) Å
*c* = 12.8444 (16) Å
β = 114.733 (2)°
*V* = 1425.7 (3) Å^3^
*Z* = 4
Mo *K*α radiation
μ = 0.09 mm^−1^
*T* = 100 (2) K
0.53 × 0.43 × 0.19 mm

#### Data collection

Bruker SMART APEX CCD diffractometer
Absorption correction: multi-scan (*SADABS*; Bruker, 2007 ▶) *T*_min_ = 0.886, *T*_max_ = 0.984
13755 measured reflections
3526 independent reflections
2941 reflections with *I* > 2σ(*I*)
*R*_int_ = 0.027

#### Refinement

*R*[*F* ^2^ > 2σ(*F* ^2^)] = 0.040
*wR*(*F* ^2^) = 0.109
*S* = 1.03
3526 reflections
193 parameters
H-atom parameters constrained
Δρ_max_ = 0.31 e Å^−3^
Δρ_min_ = −0.26 e Å^−3^

### 

Data collection: *APEX2* (Bruker, 2007 ▶); cell refinement: *SAINT* (Bruker, 2007 ▶); data reduction: *SAINT*; program(s) used to solve structure: *SHELXTL* (Sheldrick, 2008 ▶); program(s) used to refine structure: *SHELXTL*; molecular graphics: *Mercury* (Macrae *et al.*, 2006 ▶); software used to prepare material for publication: *SHELXTL*.

## Supplementary Material

Crystal structure: contains datablocks global, I. DOI: 10.1107/S1600536808033862/hb2805sup1.cif

Structure factors: contains datablocks I. DOI: 10.1107/S1600536808033862/hb2805Isup2.hkl

Additional supplementary materials:  crystallographic information; 3D view; checkCIF report

## Figures and Tables

**Table 1:** Hydrogen-bond geometry (Å, °)

*D*—H⋯*A*	*D*—H	H⋯*A*	*D*⋯*A*	*D*—H⋯*A*
N1—H1⋯O2^i^	0.88	1.99	2.8634 (13)	173
C15—H15*A*⋯O1^ii^	0.99	2.59	3.5411 (15)	161

Symmetry codes: (i) 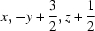; (ii) 
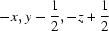
.

**Fig. 1.**
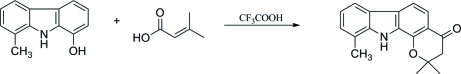
Reaction sequence

**Fig. 2.**
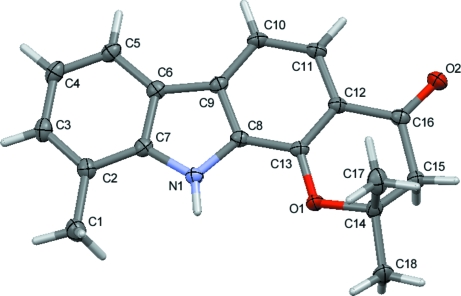
View of (I) showing xx% displacement ellipsoids. H atoms are represented in stick mode.

**Fig. 3.**
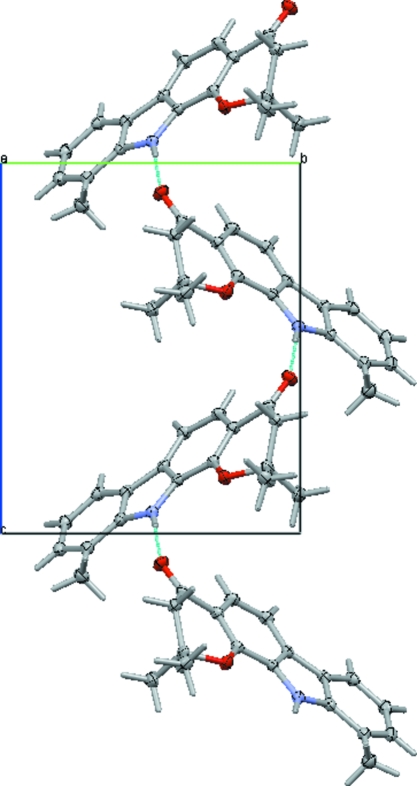
Packing view of (I) down the a axis showing chains built by the N—H···O hydrogen bonds (indicated by blue dashed lines).

**Fig. 4.**
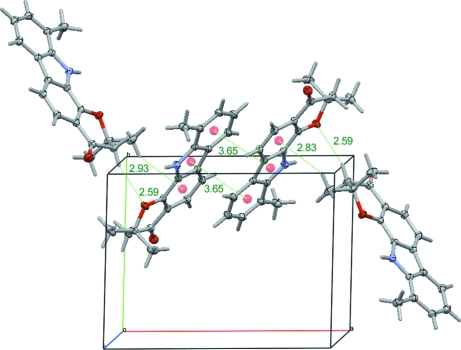
Packing view of (I) showing the secondary C—H···π and C—H···O interactions indicated by green lines. Numbers given are distances in Å. N—H···O hydrogen bonds are omitted for clarity.

## supplementary crystallographic information

### Comment

Carbazole alkaloids have been isolated from the taxonomically related higher plants of the genus Murraya, Glycosmis, and Clausena from the family Rutaceae. Among the carbazole alkaloids pyranocarbazole alkaloids play a very important role. In this class girinimbine was the first member of the pyrano[3,2-*a*]carbazole alkaloid family to be isolated from *M*. Koenigii Spreng (Knölker & Reddy, 2002, and references therein). The isolation of these classes of compounds became an active area of study since these compounds possess high levels of biological and pharmacological activity. Hence we attempted to synthesize pyranocarbazoles in a simple and efficient route.

Using trifluoroacetic acid as the acylating agent we had been able to synthezize in high yields a range of pyranocarbazolones and we recently reported (Sridharan *et al.*, 2007) the synthesis and crystallographic behaviour of 2,3-dihydro-2,2,8-trimethylpyrano[2,3-*a*]carbazol-4-(11*H*)-one. As an extension of this reasearch, and to further proof the credibility of trifluoroacetic acid as a good acylating agent, we further extended this synthetic route with a series of substituted 1-hydroxycarbazoles. The components thus synthesized were used as starting synthons to develop routes towards substituted pyranocarbazole derivatives. Herein we report the crystal structures of two of the compounds thus obtained: 2,3-dihydro-2,2,9-trimethylpyrano[2,3-*a*]carbazol-4-(11*H*)-one (Sridharan, Rajendra Prasad & Zeller, 2008), the title compound of the preceeding article in this journal) and of the title compound 2,3-dihydro-2,2,10-trimethylpyrano[2,3-*a*]carbazol-4-(11*H*)-one (Figure 1).

The single-crystal structure confirmed the formation of the dihydropyran*o*-[2,3-*a*]carbazol-4(11*H*)-one framework as shown in Figure 2. Data collection and structure refinement were unproblematic and all structural parameters (bond lengths, angles, *etc*) are in the expected ranges. The molecules crystallize in a monoclinic setting in *P*2_1_/*c* with four largely planar molecules per unit cell. The plane defined by the *sp*^2^ hybridized carbon atoms, the C1 methyl and C15 methylene carbon atoms, and the N and O atoms has an r.m.s. deviation from planarity of only 0.0754 Å. Of all the ring C atoms only C14 of the pyran C(Me)_2_ unit is significately out of plane with the atoms of the four fused rings, its deviation being 0.534 (1) Å. The pyran ring thus exhibits a half chair conformation.

One of the methyl groups of the C(Me)_2_ unit is also located close to the average plane of the molecule (C18 with a deviation of 0.125 (2) Å). The other, C17, is however located 2.039 (2) Å away from this plane and thus makes the molecule as a whole not planar and prevents it form forming extensive π-π stacked entities in the solid state. The packing is thus indeed dominated by strong N—H···O hydrogen bonds (Figure 3, Table 1) and some weaker C—H···O (Table 1, Figure 4) and C—H···π interactions (*e.g.* C18—H18*b*···*Cg*1^ii^ = 2.94 Å with *Cg*1 being the ring C8 to C13 and ii = -*x*, -1/2 + *y*, 1/2 - *z*). The only significant π···π stacking interaction with a centroid to centroid distance of 3.806 (2) Å is found between the pyrrole ring and the the aromatic ring made up of C2 to C7 (Figure 4). Two neighboring molecules related by an inversion center are forming loosly connected dimers *via* two sets of these π-π interactions (symmetry operator 1 - *x*, 2 - *y*, 1 - *z*).

The structures of the 2,2-dimethyl and the 2,2,10-methyl derivatives of the title compound are described in Sridharan, Rajendra Prasad, Ngendahimana *et al.* (2008) and Sridharan, Rajendra Prasad & Zeller (2008), the two preceeding articles in this journal. For a more detailed comparison of structures and packing of the three two derivatives please see in Sridharan, Rajendra Prasad & Zeller (2008).

### Experimental

1-hydroxy-8-methylcarbazole (0.001 mol) dissolved in 10 ml of trifluroaceticacid and was heated with 3,3-dimethylacrylicacid (0.001 mol) at 323 K for 5 h. The reaction was monitored by TLC. After completion of the reaction, the excess trifluroacetic acid was removed using rotary evaporation. The solid that precipitated out was poured onto ice water, then extracted using ethyl acetate and dried over anhydrous sodium sulfate and filtered. Then the solvent was removed under vacuum and the residue was purified by column chromatography on silica gel using petroleum ether/ethyl acetate (95:5 v/v) as eluant to yield yellow plates of (I) (0.239 g, 86%), m.p. 475–477 K.

### Refinement

All hydrogen atoms were added in calculated positions with C—H = 0.99Å (methylene), 0.95Å (aromatic) and 0.98 Å (methyl) and N—H = 0.88 Å. They were refined as riding with U_iso_(H) = 1.2U_eq_(C,N) or 1.5U_eq_(methyl C).

### Crystal data

**Table:**

C_18_H_17_NO_2_	*F*(000) = 592
*M**_r_* = 279.33	*D*_x_ = 1.301 Mg m^−^^3^
Monoclinic, *P*2_1_/*c*	Mo *K*α radiation, λ = 0.71073 Å
Hall symbol: -P 2ybc	Cell parameters from 4194 reflections
*a* = 12.9740 (16) Å	θ = 2.8–31.5°
*b* = 9.4195 (12) Å	µ = 0.09 mm^−^^1^
*c* = 12.8444 (16) Å	*T* = 100 K
β = 114.733 (2)°	Plate, yellow
*V* = 1425.7 (3) Å^3^	0.53 × 0.43 × 0.19 mm
*Z* = 4	

### Data collection

**Table:**

Bruker APEXII CCD diffractometer	3526 independent reflections
Radiation source: fine-focus sealed tube	2941 reflections with *I* > 2σ(*I*)
graphite	*R*_int_ = 0.027
ω scans	θ_max_ = 28.3°, θ_min_ = 1.7°
Absorption correction: multi-scan (SADABS; Bruker, 2007)	*h* = −17→17
*T*_min_ = 0.886, *T*_max_ = 0.984	*k* = −12→12
13755 measured reflections	*l* = −17→17

### Refinement

**Table:**

Refinement on *F*^2^	Primary atom site location: structure-invariant direct methods
Least-squares matrix: full	Secondary atom site location: difference Fourier map
*R*[*F*^2^ > 2σ(*F*^2^)] = 0.040	Hydrogen site location: inferred from neighbouring sites
*wR*(*F*^2^) = 0.109	H-atom parameters constrained
*S* = 1.03	*w* = 1/[σ^2^(*F*_o_^2^) + (0.0532*P*)^2^ + 0.5237*P*] where *P* = (*F*_o_^2^ + 2*F*_c_^2^)/3
3526 reflections	(Δ/σ)_max_ = 0.002
193 parameters	Δρ_max_ = 0.31 e Å^−^^3^
0 restraints	Δρ_min_ = −0.26 e Å^−^^3^

### Special details

**Table:**

Geometry. All e.s.d.'s (except the e.s.d. in the dihedral angle between two l.s. planes) are estimated using the full covariance matrix. The cell e.s.d.'s are taken into account individually in the estimation of e.s.d.'s in distances, angles and torsion angles; correlations between e.s.d.'s in cell parameters are only used when they are defined by crystal symmetry. An approximate (isotropic) treatment of cell e.s.d.'s is used for estimating e.s.d.'s involving l.s. planes.
Refinement. Refinement of *F*^2^ against ALL reflections. The weighted *R*-factor *wR* and goodness of fit *S* are based on *F*^2^, conventional *R*-factors *R* are based on *F*, with *F* set to zero for negative *F*^2^. The threshold expression of *F*^2^ > σ(*F*^2^) is used only for calculating *R*-factors(gt) *etc*. and is not relevant to the choice of reflections for refinement. *R*-factors based on *F*^2^ are statistically about twice as large as those based on *F*, and *R*- factors based on ALL data will be even larger.

### Fractional atomic coordinates and isotropic or equivalent isotropic displacement parameters (Å^2^)

**Table:**

	*x*	*y*	*z*	*U*_iso_*/*U*_eq_	
C1	0.30161 (12)	1.23026 (14)	0.61446 (11)	0.0287 (3)	
H1A	0.3274	1.1580	0.6749	0.043*	
H1B	0.2199	1.2193	0.5680	0.043*	
H1C	0.3168	1.3249	0.6493	0.043*	
C2	0.36377 (10)	1.21263 (12)	0.53998 (10)	0.0205 (2)	
C3	0.44870 (10)	1.30290 (13)	0.54197 (11)	0.0235 (3)	
H3	0.4698	1.3806	0.5939	0.028*	
C4	0.50503 (11)	1.28432 (13)	0.47042 (11)	0.0244 (3)	
H4	0.5636	1.3484	0.4757	0.029*	
C5	0.47647 (10)	1.17431 (13)	0.39258 (10)	0.0217 (2)	
H5	0.5142	1.1624	0.3437	0.026*	
C6	0.39071 (10)	1.08061 (12)	0.38720 (10)	0.0186 (2)	
C7	0.33727 (9)	1.09998 (12)	0.46184 (10)	0.0178 (2)	
C8	0.25796 (9)	0.90879 (12)	0.35603 (9)	0.0165 (2)	
C9	0.33879 (9)	0.95775 (12)	0.31816 (10)	0.0176 (2)	
C10	0.35259 (10)	0.88720 (13)	0.22829 (10)	0.0197 (2)	
H10	0.4078	0.9184	0.2029	0.024*	
C11	0.28477 (10)	0.77237 (13)	0.17803 (10)	0.0199 (2)	
H11	0.2932	0.7246	0.1169	0.024*	
C12	0.20236 (10)	0.72342 (12)	0.21537 (10)	0.0176 (2)	
C13	0.19004 (9)	0.79075 (12)	0.30620 (10)	0.0165 (2)	
C14	0.06708 (10)	0.60905 (12)	0.32183 (10)	0.0208 (2)	
C15	0.03119 (10)	0.58064 (13)	0.19434 (10)	0.0199 (2)	
H15A	0.0054	0.4810	0.1773	0.024*	
H15B	−0.0338	0.6428	0.1493	0.024*	
C16	0.12561 (10)	0.60623 (12)	0.15718 (10)	0.0186 (2)	
C17	0.15681 (12)	0.50413 (14)	0.39605 (11)	0.0288 (3)	
H17A	0.2229	0.5095	0.3778	0.043*	
H17B	0.1255	0.4078	0.3811	0.043*	
H17C	0.1798	0.5275	0.4771	0.043*	
C18	−0.03389 (12)	0.61299 (15)	0.35277 (12)	0.0302 (3)	
H18A	−0.0085	0.6424	0.4329	0.045*	
H18B	−0.0681	0.5183	0.3425	0.045*	
H18C	−0.0902	0.6808	0.3029	0.045*	
N1	0.25730 (8)	0.99440 (10)	0.44254 (8)	0.0176 (2)	
H1	0.2137	0.9838	0.4791	0.021*	
O1	0.11424 (7)	0.75262 (9)	0.34888 (7)	0.01967 (19)	
O2	0.13335 (7)	0.53564 (10)	0.08062 (7)	0.0245 (2)	

### Atomic displacement parameters (Å^2^)

**Table:**

	*U*^11^	*U*^22^	*U*^33^	*U*^12^	*U*^13^	*U*^23^
C1	0.0343 (7)	0.0268 (7)	0.0265 (6)	−0.0040 (5)	0.0145 (6)	−0.0059 (5)
C2	0.0223 (6)	0.0185 (5)	0.0189 (5)	0.0003 (4)	0.0068 (5)	0.0011 (4)
C3	0.0256 (6)	0.0190 (6)	0.0220 (6)	−0.0024 (5)	0.0061 (5)	0.0003 (4)
C4	0.0227 (6)	0.0229 (6)	0.0251 (6)	−0.0052 (5)	0.0076 (5)	0.0031 (5)
C5	0.0201 (6)	0.0238 (6)	0.0215 (6)	−0.0018 (5)	0.0090 (5)	0.0040 (5)
C6	0.0187 (5)	0.0185 (5)	0.0182 (5)	0.0004 (4)	0.0074 (4)	0.0025 (4)
C7	0.0173 (5)	0.0168 (5)	0.0185 (5)	0.0008 (4)	0.0068 (4)	0.0031 (4)
C8	0.0177 (5)	0.0170 (5)	0.0160 (5)	0.0018 (4)	0.0083 (4)	0.0024 (4)
C9	0.0175 (5)	0.0179 (5)	0.0182 (5)	0.0002 (4)	0.0083 (4)	0.0032 (4)
C10	0.0192 (5)	0.0229 (6)	0.0209 (6)	−0.0001 (4)	0.0122 (5)	0.0022 (4)
C11	0.0214 (6)	0.0230 (6)	0.0191 (5)	0.0014 (4)	0.0121 (5)	0.0002 (4)
C12	0.0181 (5)	0.0185 (5)	0.0182 (5)	0.0008 (4)	0.0096 (4)	0.0011 (4)
C13	0.0163 (5)	0.0176 (5)	0.0177 (5)	0.0015 (4)	0.0090 (4)	0.0024 (4)
C14	0.0255 (6)	0.0189 (6)	0.0219 (6)	−0.0073 (4)	0.0137 (5)	−0.0034 (4)
C15	0.0200 (5)	0.0218 (6)	0.0200 (5)	−0.0038 (4)	0.0104 (5)	−0.0035 (4)
C16	0.0199 (5)	0.0197 (6)	0.0177 (5)	0.0019 (4)	0.0093 (4)	0.0019 (4)
C17	0.0388 (7)	0.0219 (6)	0.0242 (6)	−0.0039 (5)	0.0117 (6)	0.0022 (5)
C18	0.0349 (7)	0.0347 (7)	0.0312 (7)	−0.0148 (6)	0.0237 (6)	−0.0103 (6)
N1	0.0196 (5)	0.0175 (5)	0.0183 (5)	−0.0013 (4)	0.0106 (4)	−0.0008 (4)
O1	0.0229 (4)	0.0189 (4)	0.0231 (4)	−0.0050 (3)	0.0155 (4)	−0.0034 (3)
O2	0.0277 (5)	0.0264 (5)	0.0239 (4)	−0.0031 (4)	0.0153 (4)	−0.0064 (3)

### Geometric parameters (Å, °)

**Table:**

C1—C2	1.4963 (18)	C11—C12	1.4195 (16)
C1—H1A	0.9800	C11—H11	0.9500
C1—H1B	0.9800	C12—C13	1.3945 (16)
C1—H1C	0.9800	C12—C16	1.4649 (16)
C2—C3	1.3836 (17)	C13—O1	1.3594 (13)
C2—C7	1.4010 (16)	C14—O1	1.4650 (13)
C3—C4	1.4040 (19)	C14—C18	1.5202 (17)
C3—H3	0.9500	C14—C17	1.5205 (18)
C4—C5	1.3787 (18)	C14—C15	1.5270 (16)
C4—H4	0.9500	C15—C16	1.5086 (16)
C5—C6	1.3991 (16)	C15—H15A	0.9900
C5—H5	0.9500	C15—H15B	0.9900
C6—C7	1.4103 (16)	C16—O2	1.2254 (14)
C6—C9	1.4420 (16)	C17—H17A	0.9800
C7—N1	1.3826 (14)	C17—H17B	0.9800
C8—N1	1.3759 (14)	C17—H17C	0.9800
C8—C13	1.3966 (16)	C18—H18A	0.9800
C8—C9	1.4057 (15)	C18—H18B	0.9800
C9—C10	1.4068 (16)	C18—H18C	0.9800
C10—C11	1.3732 (17)	N1—H1	0.8800
C10—H10	0.9500		
			
C2—C1—H1A	109.5	C13—C12—C16	118.57 (10)
C2—C1—H1B	109.5	C11—C12—C16	121.31 (10)
H1A—C1—H1B	109.5	O1—C13—C12	124.94 (10)
C2—C1—H1C	109.5	O1—C13—C8	116.73 (10)
H1A—C1—H1C	109.5	C12—C13—C8	118.31 (10)
H1B—C1—H1C	109.5	O1—C14—C18	103.62 (9)
C3—C2—C7	115.69 (11)	O1—C14—C17	108.49 (10)
C3—C2—C1	123.92 (11)	C18—C14—C17	111.68 (11)
C7—C2—C1	120.39 (11)	O1—C14—C15	109.02 (9)
C2—C3—C4	122.68 (12)	C18—C14—C15	112.03 (10)
C2—C3—H3	118.7	C17—C14—C15	111.62 (10)
C4—C3—H3	118.7	C16—C15—C14	112.81 (9)
C5—C4—C3	120.85 (11)	C16—C15—H15A	109.0
C5—C4—H4	119.6	C14—C15—H15A	109.0
C3—C4—H4	119.6	C16—C15—H15B	109.0
C4—C5—C6	118.48 (11)	C14—C15—H15B	109.0
C4—C5—H5	120.8	H15A—C15—H15B	107.8
C6—C5—H5	120.8	O2—C16—C12	123.50 (11)
C5—C6—C7	119.46 (11)	O2—C16—C15	121.14 (11)
C5—C6—C9	133.93 (11)	C12—C16—C15	115.31 (10)
C7—C6—C9	106.61 (10)	C14—C17—H17A	109.5
N1—C7—C2	127.88 (11)	C14—C17—H17B	109.5
N1—C7—C6	109.30 (10)	H17A—C17—H17B	109.5
C2—C7—C6	122.81 (11)	C14—C17—H17C	109.5
N1—C8—C13	128.38 (10)	H17A—C17—H17C	109.5
N1—C8—C9	110.17 (10)	H17B—C17—H17C	109.5
C13—C8—C9	121.44 (10)	C14—C18—H18A	109.5
C8—C9—C10	119.89 (11)	C14—C18—H18B	109.5
C8—C9—C6	105.94 (10)	H18A—C18—H18B	109.5
C10—C9—C6	134.16 (11)	C14—C18—H18C	109.5
C11—C10—C9	118.78 (10)	H18A—C18—H18C	109.5
C11—C10—H10	120.6	H18B—C18—H18C	109.5
C9—C10—H10	120.6	C8—N1—C7	107.97 (10)
C10—C11—C12	121.47 (11)	C8—N1—H1	126.0
C10—C11—H11	119.3	C7—N1—H1	126.0
C12—C11—H11	119.3	C13—O1—C14	116.68 (9)
C13—C12—C11	120.07 (11)		
			
C7—C2—C3—C4	0.25 (18)	C11—C12—C13—O1	−179.88 (10)
C1—C2—C3—C4	−179.51 (12)	C16—C12—C13—O1	2.67 (17)
C2—C3—C4—C5	0.89 (19)	C11—C12—C13—C8	1.94 (17)
C3—C4—C5—C6	−0.60 (18)	C16—C12—C13—C8	−175.51 (10)
C4—C5—C6—C7	−0.79 (17)	N1—C8—C13—O1	−1.17 (17)
C4—C5—C6—C9	179.38 (12)	C9—C8—C13—O1	−179.73 (10)
C3—C2—C7—N1	179.55 (11)	N1—C8—C13—C12	177.16 (11)
C1—C2—C7—N1	−0.67 (19)	C9—C8—C13—C12	−1.39 (16)
C3—C2—C7—C6	−1.70 (17)	O1—C14—C15—C16	53.97 (13)
C1—C2—C7—C6	178.07 (11)	C18—C14—C15—C16	168.06 (10)
C5—C6—C7—N1	−179.03 (10)	C17—C14—C15—C16	−65.85 (13)
C9—C6—C7—N1	0.84 (13)	C13—C12—C16—O2	−176.05 (11)
C5—C6—C7—C2	2.02 (17)	C11—C12—C16—O2	6.53 (18)
C9—C6—C7—C2	−178.11 (10)	C13—C12—C16—C15	6.45 (15)
N1—C8—C9—C10	−178.90 (10)	C11—C12—C16—C15	−170.97 (11)
C13—C8—C9—C10	−0.10 (17)	C14—C15—C16—O2	147.37 (11)
N1—C8—C9—C6	0.40 (12)	C14—C15—C16—C12	−35.06 (14)
C13—C8—C9—C6	179.19 (10)	C13—C8—N1—C7	−178.57 (11)
C5—C6—C9—C8	179.10 (12)	C9—C8—N1—C7	0.12 (12)
C7—C6—C9—C8	−0.74 (12)	C2—C7—N1—C8	178.28 (11)
C5—C6—C9—C10	−1.8 (2)	C6—C7—N1—C8	−0.60 (12)
C7—C6—C9—C10	178.40 (12)	C12—C13—O1—C14	18.95 (16)
C8—C9—C10—C11	1.04 (17)	C8—C13—O1—C14	−162.84 (10)
C6—C9—C10—C11	−178.01 (12)	C18—C14—O1—C13	−165.62 (10)
C9—C10—C11—C12	−0.48 (17)	C17—C14—O1—C13	75.58 (12)
C10—C11—C12—C13	−1.04 (18)	C15—C14—O1—C13	−46.16 (13)
C10—C11—C12—C16	176.35 (11)		

### Hydrogen-bond geometry (Å, °)

**Table:**

*D*—H···*A*	*D*—H	H···*A*	*D*···*A*	*D*—H···*A*
N1—H1···O2^i^	0.88	1.99	2.8634 (13)	173
C15—H15A···O1^ii^	0.99	2.59	3.5411 (15)	161

Symmetry codes: (i) *x*, −*y*+3/2, *z*+1/2; (ii) −*x*, *y*−1/2, −*z*+1/2.
